# A new flagship of global football: the rise of global attention towards Saudi Arabia’s pro league

**DOI:** 10.3389/fspor.2024.1293751

**Published:** 2024-02-01

**Authors:** Michael Mutz

**Affiliations:** Institute of Sport Science, Justus-Liebig-University Giessen, Giessen, Germany

**Keywords:** Global South, Google Trends, football leagues, World Systems Theory, public interest

## 1 Introduction

Saudi Arabian football clubs have spent a seemingly irrational amount of money in the 2023 summer transfer window to sign a number of world-class football players. Al-Nassr FC signed five times Ballon d'Or winner Cristiano Ronaldo for a rumored annual salary of 200 million Euros; Al-Hilal SFC secured the commitment of Brazilian superstar Neymar; and the French-Algerian top striker Karim Benzema joined Al-Ittihad FC. Experts are rubbing their eyes not only at the transfer fees, but also at the salaries with which the Saudi Pro League (SPL) hires renowned players. Despite the fact that transfer fees have already increased in previous years by an annual rate of >25% (1), the heated football economy has once again reached new record levels in the transfer business.

What has happened in summer 2023 is a coordinated move. To prepare for a post-oil era, Saudi Arabia needs to grow new business opportunities, not least around sports and tourism. As one pillar of its “Vision 2030” strategy, the country attempts to develop high-class entertainment opportunities, host major sporting events and reach international excellence in a number of professional sports (2). League officials stress that professional football is regarded as a growing market and that current investments aim to develop the SPL to one of the world's leading football leagues, thereby securing a substantial share in the future of the business (3). With Saudi Arabia's ambitions as big as its wallet, the question arises: Is the SPL really on its way to becoming a global flagship league?

## 2 The world system of football

To understand the global football economy, it can be insightful to take a brief look at World Systems Theory (WST) (4, 5). WST postulates that capitalist economy is best to conceive as a global system with structures and rules that establish and reproduce wealth and power hierarchies. The economic world system has a core and several layers of semi-periphery and periphery around it. While the core produces capital-intensive, high quality goods, this production relies on raw materials and labor of the periphery. Unequally distributed power allows creating trade conditions in favor of the core that help secure a surplus value and accumulate capital, while the periphery remains underdeveloped.

Scholars have applied WST to world football (6, 7) and within this theoretical framework, one can convincingly argue that the so-called European Top 5 leagues (in England, Spain, Italy, Germany, France) make up the core of the world football business. They deliver competitions of the highest sporting quality, their clubs have the most expensive squads in terms of transfer values (8), and they showcase the sport's superstars to a global audience. Global media and marketing rights alone are flushing 7.7 billion Euros p.a. into these five leagues (9). Other (mostly) European leagues are useful breeding grounds for putting talents through its paces, polish their technical and tactical skills, give them opportunities to prove themselves and then sell them to a club in a Top 5 league with a whopping profit. Portugal and Belgium are textbook examples of such “launching pad” leagues (10, 11). The periphery of world football was all too often the African and South American continent, where talent seems to abound and football boarding schools, agencies and scouts fish for the most promising diamonds in the rough (12–15). Scholars described these relations as “value chains” or global production networks (16).

The channels of player migration between core and periphery often build on cultural proximity and a shared colonial past (11). For instance, the first stop for Latin American players in Europe is often Portugal or Spain, while Belgian and French clubs often sign players from francophone Africa (11, 16). Transfer networks (i.e., the figuration of agencies, scouts, clubs and academies facilitating player migration) form along historical lines and often resemble neocolonial relations (16). Notwithstanding these cultural and historical factors, the global economic disparities make up the structural basis for football-related migration. International football transfers follow the logic of money and it was quite clear for long that financial potency was concentrated in Europe.

Scholars have also characterized globalized football as a competition of leagues and clubs for their share in a global market (17). However, before football products can generate revenue, the leagues and clubs must first become visible. The attention of fans usually relates to sporting quality and, in particular, transnational attention often focus on the best clubs, events and players in the world (18). Hence, new markets are more easily to approach when the quality of the product at stake is high. Football's superstars stand for a unique potential to create entertaining performances and are a source of identification. Given the scientific evidence that “superstar effects” exist, e.g., with regard to ticket sales (19–21), the currently signed stars should help to secure SPL a substantial share of global attention.

## 3 The rise of attention towards SPL football

To illustrate the relative increase of public attention that SPL has received in several countries, I make use of Google Trends data. These data reflect public attention to an issue or event, i.e., whether people are aware of something at all and how much they are interested in it. Normally, public attention fluctuates greatly and follows current topics and debates. Nevertheless, a previously marginalized football league can potentially also generate some lasting interest, especially if the superstars repeatedly create spectacular goals and match action that constantly stimulate interest anew. Past research has used Google Trends, for instance, to forecast the spread of influenza epidemics (22), predict private consumption patterns (23), or describe changed leisure behaviors during the COVID-19 pandemic (24). For a sports product, visibility is a necessary but not sufficient condition for commercial success. Here, awareness and attention may resemble an initial curiosity that must not (but could, of course) translate into viewership, fan loyalty, or revenues.

In the present paper, the topical search for the “Saudi Professional League” (explicitly related to the topic “football league”) is used. Topical search data include the exact phrase, but also misspellings and acronyms, and cover all languages. For cross-national comparisons, topical search data are more reliable than data on exact search terms.

**Figure 1:**
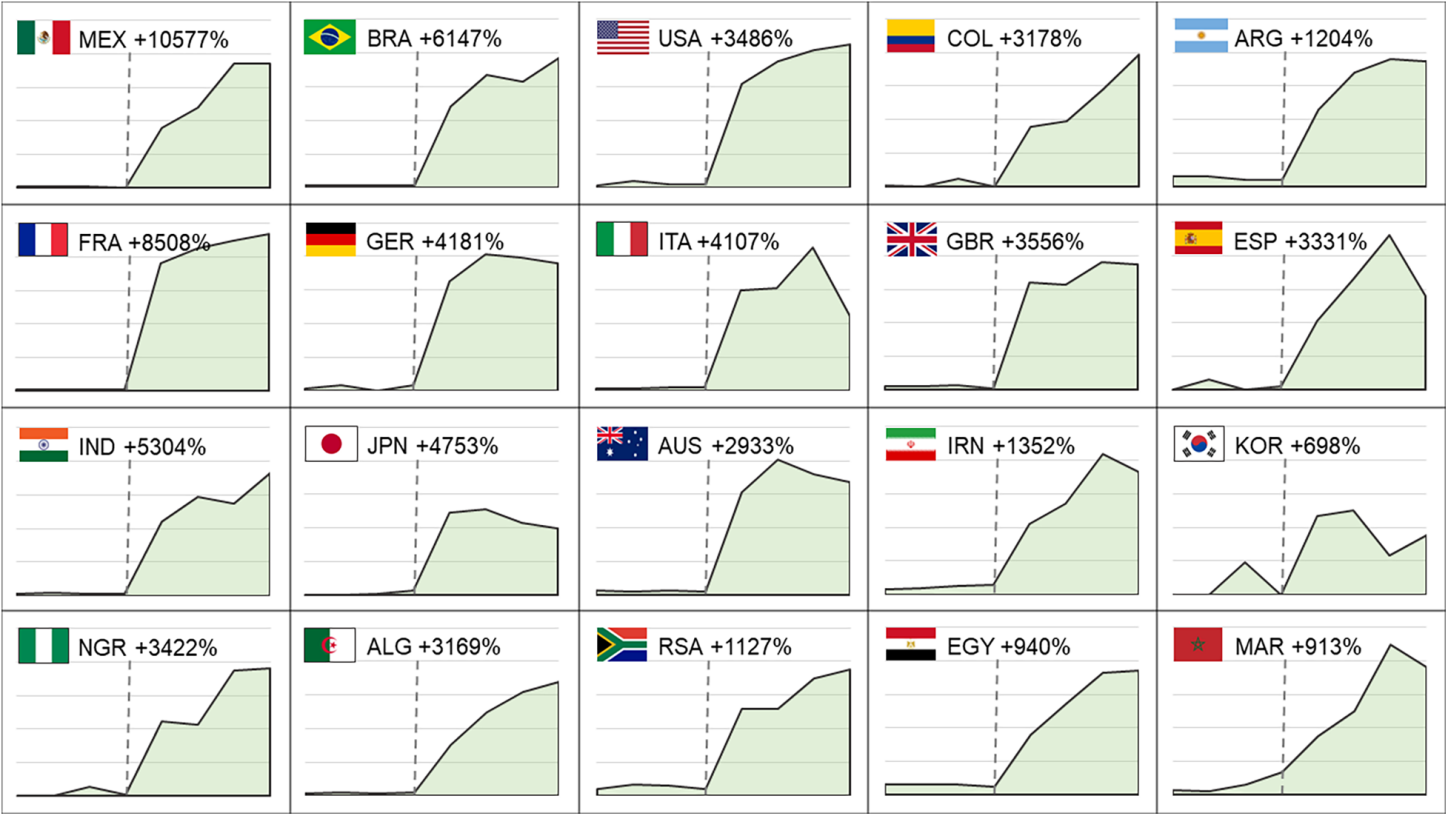
The Rise of Public Attention towards the Saudi Pro League from 2022 to 2023. The graphs show attention measured by Google Searches on the first four match days of 2022/23 season (left of dashed line) and the first four match days of 2023/24 season (right of dashed line). Percentages indicate the growth rate based on the mean levels of attention.

I illustrate the rise of public attention for a selection of 20 countries (Figure 1). These 20 countries represent different world regions; they position within the Top 100 in the FIFA Men's Ranking (25); and with few exceptions include some of the most populous countries. Google has a market share in the search engine market of >90% in all countries shown (26), except the United States (89%), Japan (75%) and South Korea (63%). These market shares make Google Trends data a good approximation of public attention in the respective countries. The figure shows the search interest in the indicated country during the first four match days of the 2022/23 season and the 2023/24 season. I only count days on which at least one league match took place, which are 14 single days in 2022 and 11 days in 2023. The growth rate indicates the rise in the mean level of search volume.

Data show that the SPL has received a massive increase in public attention throughout all countries and world regions. The highest increase in search volume is indicated for Mexico (10,577%), France (8,508%), Brazil (6,147%), and India (5,304%); the lowest increase for South Korea (698%). In the majority of countries, public attention is more than 30 times higher in 2023 compared to 2022. Although these high growth rates reflect the fact that the baseline level of public attention towards SPL football was marginal in many countries, they still indicate that the signing of superstars triggered initial interest in the league. In countries where SPL football already received some attention in 2022/23, such as Morocco, Egypt and South Africa, this level has still increased by 9–10 times in 2023/24.

## 4 The SPL’s current share of global attention

**Figure 2:**
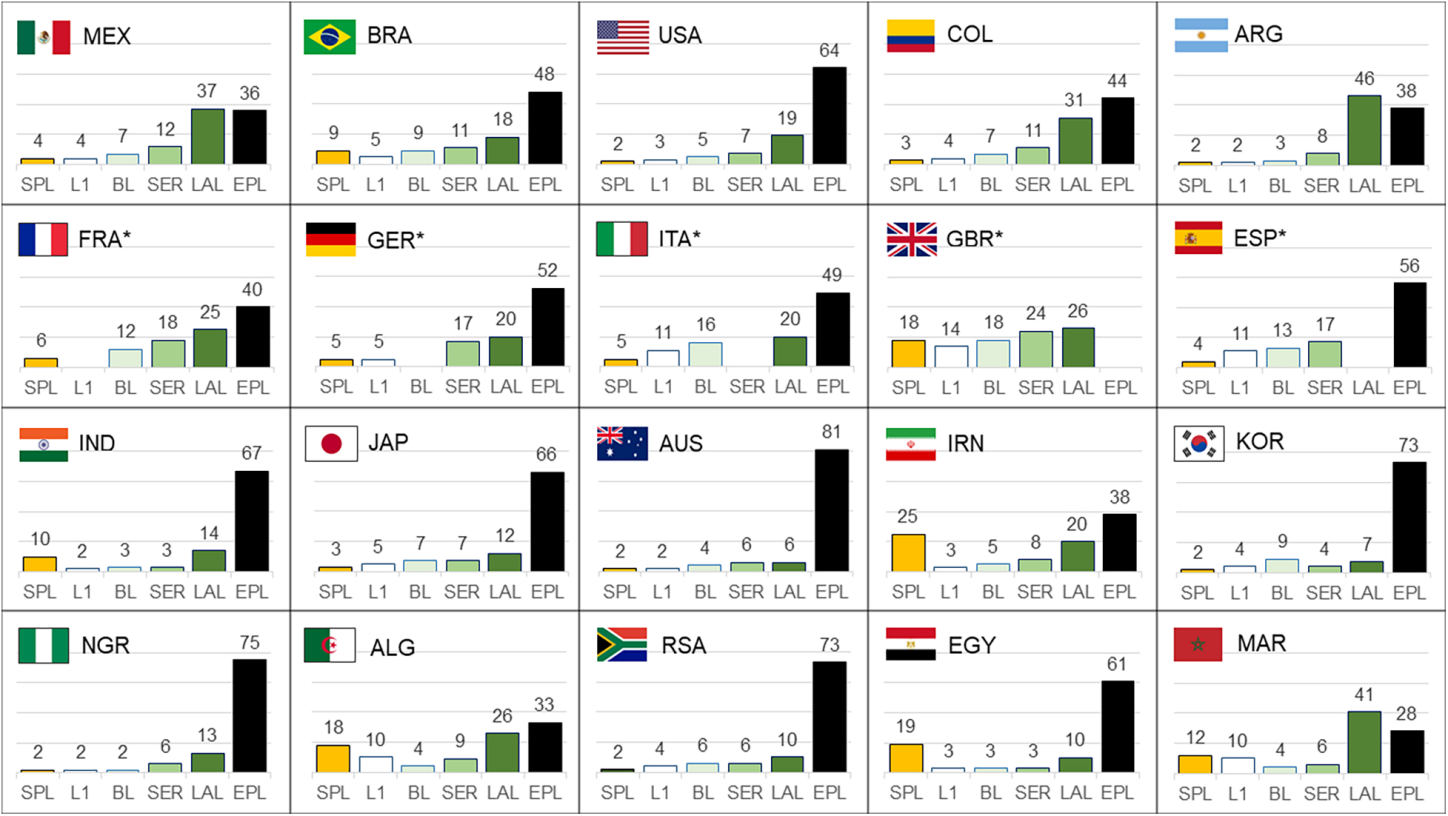
The relative level of public attention towards the Saudi Pro League (SPL) compared to the English Premier League (EPL), Spanish La Liga (LAL), Italien Serie A (SER), German Bundesliga (BL) and French Ligue 1 (L1). The graphs show attention measured by Google Searches between August 18 and September 3, 2023, which includes three match days at the start of the season. ^*^The domestic league was excluded in these countries.

A second analysis shows the proportion of attention directed towards SPL in comparison to the five major European football leagues, the English Premier League (EPL), Spanish La Liga, Italian Serie A, German Bundesliga and French Ligue 1 (Figure 2). These leagues are often considered the best in the world and thus receive considerable attention at a global level. Data show that the uncontested number one league in terms of public interest is the EPL, followed by the Spanish league. The German, Italian and French leagues receive lower levels of attention outside of their national markets. The public interest in the SPL is comparable to the level of attention the smallest of the Top 5 leagues receive. SPL has already a strong position in North African and Arab countries, such as Morocco, Algeria, Egypt, and Iran. In addition, Brazil and India are also important emerging markets where SPL received relatively high levels of attention at the start of the 2023/24 season.

## 5 Three future scenarios

These data show that the SPL generated substantial levels of public attention throughout the world at the start of the 2023/24 season. These descriptions help to conjecture how the future of the global football economy could look like. Three scenarios are most likely.

1) “Flash in the pan”. If there is no medium- or long-term perspective on how the immense investment translates into a self-sustaining business model, the project could end as quickly as it began. For the foreseeable future, Saudi Arabia is unlikely to be able to create a football culture that can produce young talent itself. Accordingly, the SPL will always have to rely on buying in talent. Unless the current investment is sustained over time, the league will not be able to establish itself on large scale. In recent years, we have already seen a similar attempt in China, where the political will aimed at making China's national football team great by strengthening the domestic league (27). Until today, however, the Chinese Super League is not a crowd pleaser outside of China and the league's strategy has changed to focus on the development of Chinese players rather than buying stars from European leagues. Hence, if the current investment is just a ‘flash in the pan’, football consumers could quickly lose interest in the SPL and the league could suffer a similar fate as the Chinese league.
2) “A world of two giants”. If the SPL succeeds in attracting more and more stars and improving the quality of its matches, it is likely that the league will be able to secure larger market shares in the football economy in the coming years. In particular, the data suggests that the league has the potential to establish itself in key emerging markets, such as Brazil, India, and the Middle East. This could mean that SPL could become profitable sooner than many of its critics think and thus become another football league with a global reach. Given the current dominance of the EPL in terms of global attention and revenue, it seems unlikely that SPL will overtake the EPL in terms of financial strength and global visibility. However, the other major leagues in today's football world, such as the German Bundesliga, Italian Serie A or French Ligue 1, are more at risk of falling behind. These leagues will certainly remain attractive on a national level, but not on a relevant global scale. As a result, the EPL and the SPL could be the two remaining giants, demanding the largest slice of the global football pie.
3) “Fragmentation along cultural lines”. Initial impressions of the global attention paid to the SPL, as well as the widespread criticism in the European media of Saudi Arabia's investment strategy, suggest that a fragmentation scenario could also emerge. While the previous scenarios assume an audience that prefers to follow the best league(s) in the world, the fragmentation scenario assumes that audiences have preferences that are also rooted in culture and values. For example, SPL attracts currently much more interest from Arab and Muslim countries than from Western countries. Western media coverage of Saudi Arabian investment in sport is highly critical (28–30), highlighting the country's repressive regime and its pre-modern values with regard to gender and sexuality, portraying the SPL as part of a “sportswashing” strategy. In contrast, press articles from the Global South take a more positive tone, highlighting celebrations at the start of the season or portray the life of star footballers in Riyadh (31–33). A politicization of football, e.g., linking political ideals to the consumption of the sport, can likely polarize audiences along value and cultural lines.

This comment shows that the SPL has received a high level of initial awareness. Whether this will translate into a commercially viable product remains to be seen. Only in the coming months and years will it become clear to what extent the league will be able to attract viewers, build fan loyalty and generate revenue. The translation of attention into followers and revenue is not trivial, but a worthwhile avenue for further research and analysis. Another limiting aspect is that Russia and China are not included in this analysis because Google's market share is too low in both countries, so that Trends data are likely biased. Hence, data do not represent two major countries and potentially important football markets of the Non-Western hemisphere.

Currently however, it seems most likely that the SPL could become an anchor point for fans in the Global South, but less so in Europe. Major League Soccer in the U.S. and the Chinese Super League could also become reference points with a trans-regional significance but without achieving truly global reach. Hence, the world system of football could become more fragmented, complex and multipolar, with more but smaller cores and several layers of overlapping periphery around them.

## References

[1] Poli R Ravenel L Besson R. Financial analysis of the transfer market in the big-5 European leagues (2010–2019). CIES Footb Observ Monthly Report. (2019) 47.

[2] Ettinger A. Saudi Arabia, sports diplomacy and authoritarian capitalism in world politics. Int J Sport Policy Politics. (2023) 15(3):531–47. 10.1080/19406940.2023.2206402

[3] The National News. Superstar Signings Key to Long-term Ambitions. (2023). Available online at: https://www.thenationalnews.com/sport/football/2023/08/31/saudi-pro-league-chief-carlo-nohra-superstar-signings-key-to-long-term-ambitions/ (accessed September 12, 2023).

[4] Wallerstein I. The Essential Wallerstein. New York: The New Press (2000). p. 471.

[5] Goldfrank WL. Paradigm regained? The rules of wallerstein’s world-system method. J World Syst Res. (2000) 11(2):150–95. 10.5195/jwsr.2000.223

[6] Brewer BD. The commercial transformation of world football and the north–south divide: a global value chain analysis. Int Rev Sociol Sport. (2019) 54(4):410–30. 10.1177/1012690217721176

[7] Finnegan L. Stepping stones? An exploration of internal football player migration in the republic of Ireland. Reg Stud Reg Sci. (2019) 6(1):596–606. 10.1080/21681376.2019.1685905

[8] Gerhards J Mutz M. Who wins the championship? Market value and team composition as predictors of success in the top European football leagues. Eur Soc. (2017) 19(3):223–42. 10.1080/14616696.2016.1268704

[9] Statista. Revenue from Broadcasting Rights of European Soccer Leagues from in 2019/20. (2021). Available online at: https://www.statista.com/statistics/627306/broadcasting-big-five-european-football-league-revenues/ (accessed September 12, 2023).

[10] Nolasco C. Player migration in Portuguese football: a game of exits and entrances. Soccer Soc. (2019) 20(6):795–809. 10.1080/14660970.2017.1419470

[11] Poli R. Migrations and trade of African football players: historic, geographical and cultural aspects. Afr Spectr. (2006) 41(3):393–414.

[12] Poli R. Understanding globalization through football: the new international division of labour, migratory channels and transnational trade circuits. Int Rev Sociol Sport. (2010) 45(4):491–506. 10.1177/1012690210370640

[13] Darby P. Moving players, traversing perspectives: global value chains, production networks and Ghanaian football labour migration. Geoforum. (2013) 50(C):43–53. 10.1016/j.geoforum.2013.06.009

[14] Darby P. Out of Africa: the exodus of elite African football talent to Europe. WorkingUSA. (2007) 10(4):443–56. 10.1111/j.1743-4580.2007.00175.x

[15] Alvito M. Our piece of the pie: brazilian football and globalization. Soccer Soc. (2007) 8(4):524–44. 10.1080/14660970701440824

[16] Darby P Esson J Ungruhe C. African Football Migration. Manchester: Manchester University Press (2022). p. 268.

[17] Hinson RE Osabutey E Kosiba JP Asiedu FO. Internationalisation and branding strategy: a case of the English premier league’s success in an emerging market. Qual Mark Res. (2020) 23(4):747–66. 10.1108/QMR-12-2017-0188

[18] Mutz M. Transnational public attention in European club football: current trends and driving forces. Eur Soc. (2015) 17(5):724–46. 10.1080/14616696.2015.1118519

[19] Brandes L Franck E Nüesch S. Local heroes and superstars: an empirical analysis of star attraction in German soccer. J Sports Econom. (2008) 9(3):266–86. 10.1177/1527002507302026

[20] Jewell RT. The effect of marquee players on sports demand: the case of U.S. major league soccer. J Sports Econom. (2017) 18(3):239–52. 10.1177/1527002514567922

[21] Humphreys BR Johnson C. The effect of superstars on game attendance: evidence from the NBA. J Sports Econom. (2020) 21(2):152–75. 10.1177/1527002519885441

[22] Ginsberg J Mohebbi MH Patel RS Brammer L Smolinski MS Brilliant L. Detecting influenza epidemics using search engine query data. Nature. (2009) 457(7232):1012–4. 10.1038/nature07634 19020500

[23] Vosen S Schmidt T. Forecasting private consumption: survey-based indicators vs. Google trends. J Forecast. (2011) 30(6):565–78. 10.1002/for.1213

[24] Abay KA Tafere K Woldemichael A. Winners and losers from COVID-19: global evidence from google search. World Bank Policy Res Work Pap Series. (2020):9268.

[25] FIFA. Mens Ranking. (2023). Available online at: https://www.fifa.com/fifa-world-ranking (accessed September 12, 2023).

[26] StatCounter. Search Engine Market Share Worldwide. (2023). Available online at: https://gs.statcounter.com/search-engine-market-share/ (accessed September 12, 2023).

[27] Peng Q Skinner J Houlihan B. An analysis of the Chinese football reform of 2015: why then and not earlier. Int J Sport Policy Politics. (2019) 11(1):1–18. 10.1080/19406940.2018.1536075

[28] Foreign Policy. Saudi Arabia Really wants you to Think It’s Cool. (2023). Available online at: https://foreignpolicy.com/2023/09/04/saudi-arabia-golf-soccer-mma-sportswashing-culture-human-rights-neom/ (accessed September 12, 2023).

[29] Der Spiegel. LGBTQ+-Community von Liverpools Ex-Kapitän Henderson Enttäuscht. (2023). Available online at: https://www.spiegel.de/sport/fussball/jordan-henderson-ex-liverpool-profi-fuer-wechsel-nach-saudi-arabien-scharf-kritisiert-a-495d1297-522a-4893-b3b5-37d021b8078b (accessed September 12, 2023).

[30] The Guardian. Premier League and Fifa Helpless Against Saudi Juggernaut’s Relentless Progress. (2023). Available online at: https://www.theguardian.com/football/blog/2023/jul/29/premier-league-fifa-helpless-against-saudi-pro-league-juggernaut (accessed September 12, 2023).

[31] The Times of India. Neymar gets Rapturous Welcome at Big-spending Saudi Club. (2023). Available online at: https://timesofindia.indiatimes.com/sports/football/top-stories/neymar-gets-rapturous-welcome-at-big-spending-saudi-club/articleshow/102863113.cms (accessed September 12, 2023).

[32] O Globo. Chegada de Astros do Futebol à Arábia Saudita Expõe Vida em ‘Bolha’ de Estrangeiros e Outro Lado da Sociedade Local. (2023). Available online at: https://oglobo.globo.com/esportes/futebol-internacional/noticia/2023/08/27/chegada-de-astros-do-futebol-a-arabia-saudita-expoe-vida-em-bolha-de-estrangeiros-e-outro-lado-da-sociedade-local.ghtml (accessed September 12, 2023).

[33] Al Jazeera. Why has Saudi Sovereign Fund Taken Over Kingdom’s Football Clubs? (2023). Available online at: https://www.aljazeera.com/sports/2023/6/8/why-has-saudi-sovereign-wealth-fund-taken-over-football-clubs (accessed September 12, 2023).

